# Forecasting malaria: ensemble modelling and predicting the impact of interventions

**DOI:** 10.1186/1475-2875-11-S1-P78

**Published:** 2012-10-15

**Authors:** Melissa Penny, Tom Smith

**Affiliations:** 1 Swiss Tropical and Public Health Institute, Basel, CH-4002, Switzerland; 2 University of Basel, Basel, CH-4003, Switzerland

## Background

Over the past decade many new mathematical models for malaria have been developed to assess the impact of interventions. Given sufficient data to inform them, these models can be useful tools for policy planning and forecasting the state of malaria in an affected region or to investigate important questions pertaining to controlling and eliminating malaria. But how accurate are these models? And importantly, how do the predictions from different models compare, and what are the uncertainties surrounding their predictions?

## Materials and methods

We present the Swiss TPH malaria modelling group's initial approach for combining and evaluating a small ensemble of models. The ensemble comprises 14 individual-based stochastic simulation models of P. falciparum dynamics, with varied assumptions about immune decay, transmission heterogeneity, and access to treatment. Models parameters were fit to an extensive library of field data.

We present a principled methodology for selecting and using an ensemble of models, which takes the accuracy and uncertainty of single-model predictions into account. The accuracy of individual models, evaluated against clinical data, is used to weight their predictions. The methodology can be applied to an ensemble composed of structural different models, potentially combining the Swiss TPH models with models from other groups. The uncertainty of the stochastic models in the ensemble is used to derive a range of reasonable values for model outcomes, such as predictions of the impact of interventions.

## Results

The proposed methodology has been piloted with an ensemble of Swiss TPH models to investigate the pre-erythrocytic vaccine RTS,S. Clinical trial data informs the models of the vaccine profile and the ensemble provides predictions of the impact of a pre-erythrocytic vaccine when scaled to national levels.

## Conclusions

This work highlights the requirements, advantages and challenges in using ensemble models for prediction of Malaria. In particular the methodology enables presentation of uncertainty in results, and to predict the health impact of new interventions where their precise influence in the model is unknown but can be discerned from clinical field trials. The methodology is applicable not only to the Swiss TPH models but could be used to make predictions in conjunction with other models.

